# Supplementary material for: Flexibility in reaction time analysis: many roads to a false positive?
Source: R Soc Open Sci. 2020 Feb 5;7(2):190831. doi: 10.1098/rsos.190831 (PMC7062108; doi:10.1098/rsos.190831)
Supplement: Supplementary Data [file rsos190831supp1.docx]

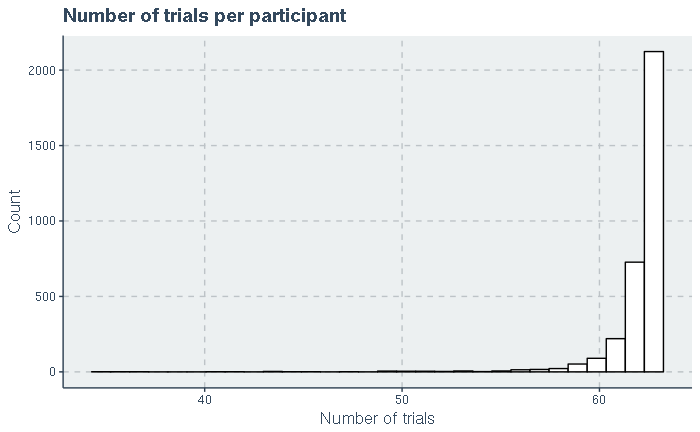

Figure S1. **Number of trials per participant in the Stroop dataset before trimming**.


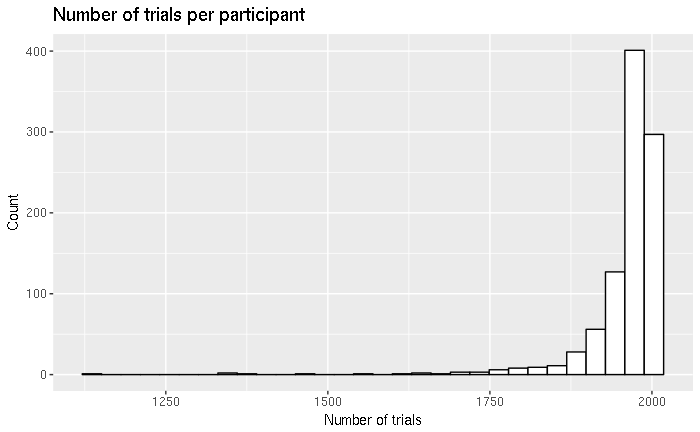


Figure S2. **Number of trials per participant in the Flexicon dataset before trimming**.

# Links to real datasets used in the simulations

## Stroop:

<https://mfr.osf.io/render?url=https://osf.io/m5qwu/?action=download%26mode=render>.

## Flexicon:

<https://figshare.com/articles/Reaction_times_and_other_skewed_distributions_problems_with_the_mean_and_the_median/6911924>

# Informal sample of articles

To confirm that *t*-tests and analyses of variance were still common methods in reaction time research we retrieved the last 50 articles published in *Frontiers in Psychology* that analyzed reaction times. To find these articles we searched in Scopus for articles published in *Frontiers in Psychology* that contained the text “reaction time”. Then articles were sorted in alphabetical order according to their title. Firstly, we checked that the paper analyzed reaction times, if not the paper was excluded from the sample, secondly what type of analysis was performed (*t*-test/ANOVA or other). This process continued until 25 articles were included in the sample. Out of the 25 articles, 19 used ANOVAs or *t*-tests on RT data.
